# Identification and validation of a tyrosine metabolism-related prognostic prediction model and characterization of the tumor microenvironment infiltration in hepatocellular carcinoma

**DOI:** 10.3389/fimmu.2022.994259

**Published:** 2022-10-20

**Authors:** Yangying Zhou, Xuanxuan Li, Guo Long, Yongguang Tao, Ledu Zhou, Jianing Tang

**Affiliations:** ^1^ Department of Oncology, Xiangya Hospital, Central South University, Changsha, China; ^2^ National Clinical Research Center for Geriatric Disorders, Xiangya Hospital, Central South University, Changsha, China; ^3^ Department of Liver Surgery, Xiangya Hospital, Central South University, Changsha, China; ^4^ Key Laboratory of Carcinogenesis and Cancer Invasion, Ministry of Education, Department of Pathology, Xiangya Hospital, Central South University, Changsha, China; ^5^ Key Laboratory of Carcinogenesis of the Ministry of Health, Cancer Research Institute, School of Basic Medicine, Central South University, Changsha, China; ^6^ Department of Thoracic Surgery, Hunan Key Laboratory of Tumor Models and Individualized Medicine, Second Xiangya Hospital, Central South University, Changsha, China

**Keywords:** hepatocellular carcinoma, tyrosine metabolism, tumor microenvironment, immunotherapy, prognosis model

## Abstract

**Background:**

Hepatocellular carcinoma (HCC) is an aggressive and heterogeneous disease characterized by high morbidity and mortality. The liver is the vital organ that participates in tyrosine catabolism, and abnormal tyrosine metabolism could cause various diseases, including HCC. Besides, the tumor immune microenvironment is involved in carcinogenesis and can influence the patients’ clinical outcomes. However, the potential role of tyrosine metabolism pattern and immune molecular signature is poorly understood in HCC.

**Methods:**

Gene expression, somatic mutations, copy number variation data, and clinicopathological information of HCC were downloaded from The Cancer Genome Atlas (TCGA) database. GSE14520 from the Gene Expression Omnibus (GEO) databases was used as a validation dataset. We performed unsupervised consensus clustering of tyrosine metabolism-related genes (TRGs) and classified patients into distinct molecular subtypes. We used ESTIMATE algorithms to evaluate the immune infiltration. We then applied LASSO Cox regression to establish the TRGs risk model and validated its predictive performance.

**Results:**

In this study, we first described the alterations of 42 TRGs in HCC cohorts and characterized the clinicopathological characteristics and tumor microenvironmental landscape of the two distinct subtypes. We then established a tyrosine metabolism-related scoring system and identified five TRGs, which were highly correlated with prognosis and representative of this gene set, namely *METTL6, GSTZ1, ADH4, ADH1A*, and *LCMT1*. Patients in the high-risk group had an inferior prognosis. Univariate and multivariate Cox proportional hazards regression analysis also showed that the tyrosine metabolism-related signature was an independent prognostic indicator. Besides, receiver operating characteristic curve (ROC) analysis demonstrated the predictive accuracy of the TRGs signature that could reliably predict 1-, 3-, and 5-year survival in both TCGA and GEO cohorts. We also got consistent results by performing clone formation and invasion analysis, and immunohistochemical (IHC) assays. Moreover, we also discovered that the TRGs signature was significantly associated with the different immune landscapes and therapeutic drug sensitivity.

**Conclusion:**

Our comprehensive analysis revealed the potential molecular signature and clinical utilities of TRGs in HCC. The model based on five TRGs can accurately predict the survival outcomes of HCC, improving our knowledge of TRGs in HCC and paving a new path for guiding risk stratification and treatment strategy development for HCC patients.

## Introduction

Hepatocellular carcinoma (HCC) is one of the most common digestive system malignancies that endanger human health. According to the latest research data released by the World Health Organization (WHO), HCC ranks the sixth most common and a third of mortality in all tumors worldwide (1, 2). The onset of HCC is usually indetectable and characterized by rapid progression, frequent metastasis, high recurrence, and poor prognosis (3). Even with the improvement of major clinical interventions, including surgery, drug-targeted therapy, radiotherapy, chemotherapy, immunotherapy, and transplantation, its prognosis remains poor, and the 5-year overall survival is less than 20% (4–6). Therefore, it is urgently needed to identify the novel molecular markers and develop a prognostic model to stratify and customize a therapeutic strategy for patients with HCC.

Tyrosine is an aromatic amino acid required for protein synthesis in all organisms, and alternative energy for molecular functions. The liver is the primary organ that participates in tyrosine catabolism. Five enzymatic reactions catalyze the tyrosine degradation. It has been reported that the disturbance of tyrosine metabolism could cause a variety of diseases like Huntington’ ‘s disease (7) and phenylketonuria (PKU) (8), as well as some cancers, including gastroesophageal malignancy (9) and lung cancer (10). Besides, previous studies also demonstrate that patients who suffered from hereditary tyrosinemia are more likely to develop HCC (11, 12). In HCC patients, the serum tyrosine is frequently upregulated (13, 14), indicating an imbalance tyrosine metabolic process in HCC. Nevertheless, poorly is understood the molecular alteration and profile of tyrosine catabolism in the development and progression of HCC.

Emerging evidence also indicates a crosstalk between tyrosine metabolism and the tumor immune microenvironment (15, 16). The tumor microenvironment (TME) plays a critical role in cancer development and clinical outcome (17). Besides the cancer cells, TME also comprises diverse cell types, including endothelial cells, immune cells, fibroblasts, and inflammatory cells, and extracellular components (growth factors, cytokines, hormones, etc.). Through the circulation and lymphatic system, malignant cells interact with neighboring cells and induce immune tolerance by releasing cell signaling molecules. In addition, tumor-infiltrating immune cells (TIICs) within the TME can also influence cancer progression (18). Currently, the majority of studies reveal only one or two tyrosine metabolism-related genes (TRGs) and cell types, while numerous genes interactions determine the antitumor impact. Therefore, understanding the characteristics of TME cell infiltration mediated by multiple TRGs may provide insights into the underlying mechanism of HCC tumorigenesis and immune response prediction.

In this study, we extensively analyzed the HCC datasets from The Cancer Genome Atlas (TCGA) and Gene Expression Omnibus (GEO) database to explore the expression patterns of TRGs and obtained an intratumoral immune landscape. We first stratified 371 patients with HCC into two distinct subtypes based on the levels of TRGs expression and prognosis. Furthermore, we established a scoring system for predicting survival outcomes and characterizing the immune landscape of HCC. Additionally, in combination with clinicopathological characteristics, our gene signature showed improved risk stratification and therapeutic predictive power for HCC, which provide new insights into precision and individualized medicine.

## Methods

### HCC data acquisition and processing

We downloaded gene expression (fragments per kilobase million, FPKM), the somatic mutations, copy number variation data and prognostic and clinicopathological information on HCC from the TCGA database. GSE14520 from the GEO database was used for the subsequent validation. After obtaining the raw microarray intensity “CEL” files, we adjusted the background and normalized the quantiles by using Robust Multichip Average. An average standard deviation of 1 was used to scale the RNA expression data. Gene expression of 424 samples (50 normal and 374 tumor samples) from 371 patients were downloaded from TCGA. The clinicopathological characteristics of the 371 HCC patients were summarized in **Supplementary Table 1**. All the analyses were performed with R (version 4.1.2) with R Bioconductor packages.

### Consensus clustering of tyrosine metabolism-related genes

We retrieved a total of forty-two TRGs from the MSigDB (KEGG_TYROSINE_METABOLISM). Using the R package “ConsensusClusterPlus,” we performed unsupervised consensus clustering of TRGs and classified patients into distinct molecular subtypes. The clustering was conducted using the following criteria: first, the cumulative distribution function (CDF) curve upgraded steadily and smoothly. Second, each group had a sufficient sample size. Lastly, the correlation of intra-group correlations increased by clustering, whereas inter-group correlations descended.

### Functional enrichment analysis

To explore the potential mechanism underlying two tyrosine metabolism subtypes involved in HCC, we performed the Gene Ontology (GO) enrichment and Kyoto Encyclopedia of Genes and Genomes (KEGG) pathway analysis using “clusterprofler” R package (19). A p<0.05 and q<0.05 were set as the thresholds. We then collected 50 gene sets of cancer hallmark-related pathways from the Gene Set Enrichment Analysis (GSEA) database. Meanwhile, the “GSVA” package was used to calculate the enrichment score of correlated pathways. The gene set “c2.cp.kegg.v7.4.symbols.gmt” was also downloaded from the MSigDB and performed as the reference gene set.

### Construction of a TRGs-based prognostic model

Least Absolute Shrinkage and Selection Operator Regression (LASSO) is a form of penalized regression that can be used to screen variables from high-dimensional data to build a prognostic model (20). In this study, we excluded patients with incomplete survival information. Then we filtered the significant Tyrosine metabolism-related genes from HCC specimens and performed the optimum survival cutoff analysis by using the “surv_cutpoint” function of the “survminer” R package. Then, we used the LASSO method in a Cox regression model to pick out the most useful prognostic genes by using the R package “glmnet”. After that, a tyrosine metabolism-related scoring system for HCC patients was established by the combination of the expression of genes and the estimated Cox regression coefficient: tyrosine metabolism-related risk score =∑^​^(coefficient of gene* expression of a gene)

According to the best cutoff risk score, HCC Patients were divided into high-risk and low-risk groups and then subjected to the Kaplan-Meier (KM) survival analysis.

### Characterization of the immune signature of HCC

We utilized the ESTIMATE algorithm to estimate the abundance of the immune cell between high-risk and low-risk groups using expression data from the TCGA database. In addition, we determined the immune cell infiltration levels in the HCC TME by using a single sample gene set enrichment analysis (ssGSEA) algorithm (21). We also analyzed the associations of PD-1 and PD-L1 expression between two subtypes.

### Drug sensitivity analysis

Broad Institute’s Cancer Cell Line Encyclopedia (CCLE) project (22) (https://portals.broadinstitute.org/ccle/) contained the RNA expression profiles of 1019 cancer cell lines, of which included 16 different liver cancer cell lines. The drug sensitivity of cancer cell lines was retrieved from the Genomics of Drug Sensitivity in Cancer (GDSC2, https://www.cancerrxgene.org/) (23), which contained 809 cell lines and sensitivity data for 198 chemicals. Lower 50% inhibiting concentration (IC50) values suggested enhanced susceptibility to compound responses. Utilizing the “oncoPredict” R package, we calculated the TCGA-LIHC cohort’s medication susceptibility.

### RNA interference

The following small interfering RNAs were purchased from Ruibo Biotechnology Co., Ltd. (Guangzhou, China): METTL6 (siG000131965A-1-5), GSTZ1(siB0006543A-1-5), ADH4 (siG000000127A-1-5), ADH1A (siG151029041002-1-5), and LCMT1 (siG000051451A-1-5). The manufacturer’s recommendations were followed for siRNA transfection when using Lipofectamine 2000 (Invitrogen, Carlsbad, CA, USA). Hep3B cells were seeded to be 70%–90% confluent at transfection. Then, 5 µl of Lipofectamine 2000 reagent and 5 µl of siRNA (10 mM) were mixed in 250 µl of OptiMEM medium. After 10 minutes of incubation at room temperature, the mixture was put dropwise into a culture dish containing 1 ml of the medium. After that, normal culture conditions (5% CO_2_, 37°C) were used to cultivate the transfected cells for 24 hours and the cells were then digested and resuspended for further experiments.

### Colony formation and invasion analysis

The cells were treated with the siRNAs for 24 hours, digested, and seeded onto six-well plates at a density of 1,000 cells per well for the colony formation experiment. After fourteen days of incubation, the cells were fixed with 4% paraformaldehyde and stained with 0.5% crystal violet. Transwell plates with an 8-mm pore for polycarbonate membrane were used to measure the ability of cells to invasion (Corning, USA). Briefly, Matrigel-coated top chambers were seeded with 5x10^5^ cells that had been suspended without serum (BD BioCoat, USA). The bottom chambers were placed with 600 µl of complete medium. The cells on the bottom side of the pore membrane were fixed and stained with crystal violet after being left alone for 24 hours.

### Immunohistochemistry study

Tissue Microarray (TMA) contains 97 samples of liver cancer tissues (n=77) and normal liver tissues (n=20) from Xiangya Hospital. Briefly, the TMA slices were first deparaffinized, followed by antigen retrieval in citrate buffer (pH 6.0) and endogenous peroxidase activity inhibition in 0.3% H_2_O_2_. The relevant primary and secondary antibodies were continually incubated with the slides until peroxidase and 3,3′-diaminobenzidine tetrahydrochloride were used for visualization. Two pathologists blindly measured the expression of METTL6, GSTZ1, ADH4, ADH1A, and LCMT1 in the liver cancer tissues from the tissue microarray using the previously described histochemical score (H-score) (24, 25). The primary antibodies were METTL6 (Proteintech, China, 16527-1-AP), GSTZ1 (Proteintech, China, 14889-1-AP), ADH4 (Proteintech, China, 16474-1-AP), ADH1A (Sigma-Aldrich, USA, HPA047814), and LCMT1 (Novus Biologicals, USA, OTI2C9).

The Human Protein Atlas (HPA) (https://www.proteinatlas.org/) is a program that integrates diverse omics technologies to map human proteins in tissues, cells, and organs (26, 27). From the tissue atlas and pathology atlas in the HPA database, we respectively got representative immunohistochemistry results for the five target proteins in HCC tumors and normal tissues.

### Statistical analysis

We performed a Chi-Square test for the analysis of differences between the two groups. The prognostic significance was evaluated by Kaplan-Meier curve analysis, including overall survival (OS), disease-specific survival (DSS), disease-free interval (DFI), progression-free interval (PFI), and relapse-free survival (RFS) with a two-tailed log-rank test. Multivariate Cox regression analysis was performed by using the R package “survival” to evaluate the role of Tyrosine metabolism-related score in the prognostic model of HCC. We used Pearson’ s correlation analyses to measure the degree of correlation between certain variables. All statistical analyses were performed using R software (version 4.1.2) and p< 0.05 was considered statistically significant.

## Results

### The genetic variation and expression landscape of TRGs in HCC

**Figure 1:**
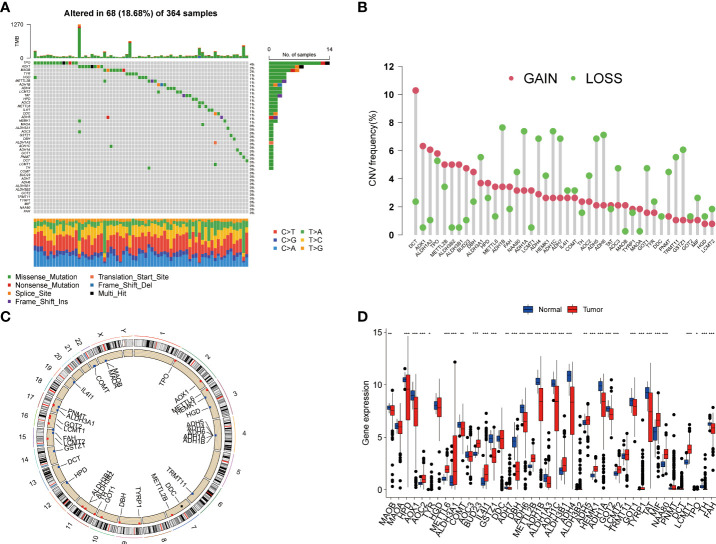
Genetic variations and transcriptional expression of TRGs in HCC. **(A)** The distribution and mutation frequencies of 42 TRGs in the TCGA HCC cohort. **(B)** Frequencies of CNV alterations of TRGs in HCC. The height of the column represents the alteration frequency. **(C)** Locations of CNV alterations in TRGs on chromosomes. **(D)** Expression distributions of 44 TRGs between HCC tumor and normal tissues. *p < 0.05, **p < 0.01, ***p < 0.001. TRGs, tyrosine metabolism-related genes; HCC, hepatocellular carcinoma; TCGA, The Cancer Genome Atlas; CNV, copynumber variation.

In this study, we included a total of 42 TRGs. Of the 364 HCC patients, genetic mutations of TRGs were found in 18.68% (68/364) of them (**Figure 1A**). Among these genes, *TPO* was the gene with the highest mutation rate, followed by *AOX1*, and *MAOB*. We also observed that missense mutation was the most frequent variant type, and C > T and C > A ranked the top single nucleotide variation (SNV) class. Then, we investigated the somatic copy number variations (CNVs) of TRGs and discovered universal copy number alterations across 42 TRGs. Of them, *DCT, AOX1, ALDH1A3, ALDH3B1, ALDH3B1* and *BUD23* showed widespread CNVs amplifications, while most of the TRGs had CNVs depletion (**Figure 1B**). **Figure 1C** presented the location of CNV alterations of Tyrosine metabolism-related genes on chromosomes. We further explored whether the genetic alterations could affect the gene expression patterns. By comparing the expression level between HCC tumors and normal tissues, we found that the TRGs expression levels were positively correlated with the CNV alterations. The TRGs with CNVs gain, like *BUD23* and *ALDH3B1*, were increased in HCC patients, while the TRGs with CNVs loss were decreased in HCC samples (**Figure 1D**). Whereas some TRGs showed opposite or no difference in CNVs alterations and expression level, indicating a complex process in the regulation of gene expression.

### Identification of tyrosine metabolism subtypes in HCC

**Figure 2:**
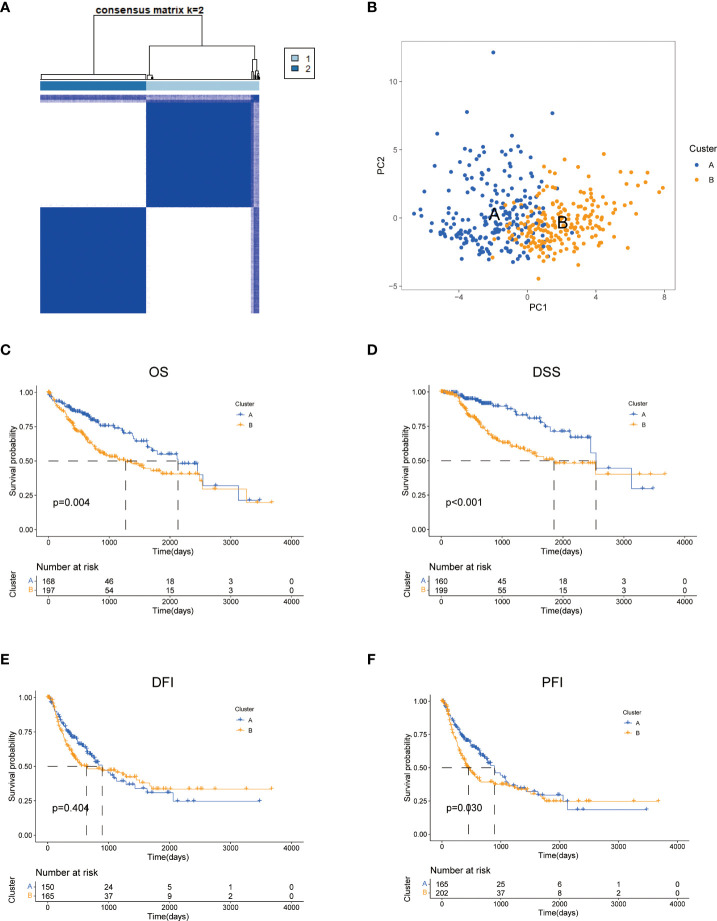
Characteristics of two TRGs subtypes divided by consistent clustering. **(A)** Consensus heatmap matrix and correlations areas of two clusters (k = 2). **(B)** PCA analysis demonstrates a distinctive difference between the two clusters. Univariate analysis shows 44 TRGs related to the OS **(C)**, the DSS **(D)**, the DFI **(E)**, and the PFI **(F)**. OS, overall survival; DSS, disease-specific survival; DFI, disease-free interval; DFI, progression-free interval.

To further explore the profile and characteristics of 42 tyrosine metabolism-related genes in HCC, we applied a consensus clustering algorithm to categorize the HCC patients based on the expression of 42 TRGs. To obtain the optimal clustering number (k value), we calculated the consistency coefficient and found that k = 2 was a preferable selection for sorting the entire cohort into Clusters A (n = 169) and B (n = 202) (**Figure 2A**). The principal component analysis (PCA) demonstrated that HCC patients were well distributed into two clusters (**Figure 2B**). The Kaplan-Meier survival analysis revealed that Cluster A had a superior OS (log-rank test, p =0.004), DSS (log-rank test, p<0.001) and PFI (log-rank test, p =0.03) among HCC patients, while the DFI between two subtypes was not statistically significant (**Figures 2C–F**).

**Figure 3:**
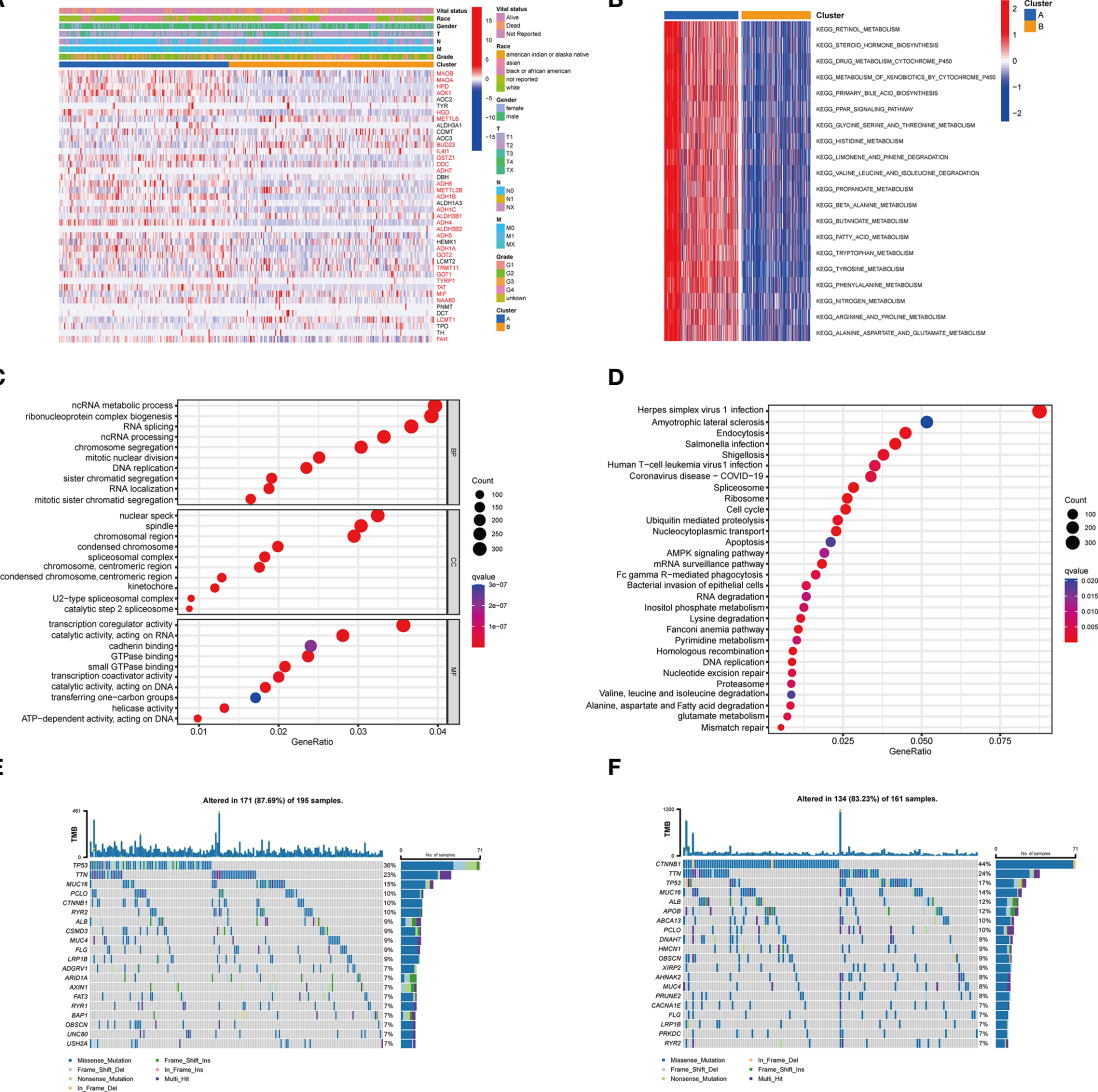
Clinicopathological features, enrichment analysis and mutation landscape of two TRGs clusters. **(A)** Differences in clinicopathologic characteristics and expression levels of TRGs between the two subtypes. **(B)** GSVA of biological pathways between two subtypes, in which blue inhibited and red represent activated pathways, respectively. **(C)** GO enrichment analysis shows the BP, CC, and MF of two TRGs subtypes. **(D)** The bubble plot depicted the KEGG pathway enrichment analysis of the two clusters. **(E)** Mutation landscape of TRGs cluster **(A–F)** Mutation landscape of TRGs cluster **(B)** GSVA, gene set variation analysis; GO, gene ontology; BP, biological process; CC, cellular component; MF, molecular function; KEGG, Kyoto Encyclopedia of Genes and Genomes; TRGs, tyrosine metabolism-related genes.

Next, we compared the two subgroups’ clinicopathological features and tyrosine metabolism-related gene expression. Some TRGs were highly expressed in Cluster A, such as *MAOB*, *MAOA*, *HPD*, and *AOX1*, while some TRGs, including *METTL6*, *BUD23*, *METTL2B*, and *LCMT1* were overexpressed in cluster B (**Figure 3A**). Moreover, we performed functional enrichment analysis to investigate the biological behavior of TRGs. GSVA enrichment analysis revealed that subtype A was significantly enriched in various substances’ metabolism (**Figure 3B**). The biological process (BP) indicated the enrichment function of the RNA metabolic process and ribonucleoprotein complex biogenesis. The cellular component (CC) showed that the TRGs were primarily correlated with nuclear speck, spindle and chromosomal region. For molecular function (MF), the transcription coregulator activity, catalytic activity, and cadherin binding were mainly enriched for the TRGs (**Figure 3C**). Moreover, the pathway analysis implied that these genes were frequently involved in the cancer-related pathways, various virus infections and substance metabolism (**Figure 3D**).

Furthermore, we explored the genetic alterations between the two subtypes. In Cluster A, we found a relatively high mutation frequency with 171 of 195 (87.69%) samples (**Figure 3E**). Among them, *TP53* had the highest mutation frequency (36%), followed by *TTN* (23%) and *MUC16* (15%). Compared to Cluster A, the Cluster B cohort demonstrated a lower mutation frequency (134 of 161 samples, 83.23%). Differentially, *CTNNB1* (44%) was the most frequently mutated gene, following *TTN* (24%) and *TP53* (17%) (**Figure 3F**).

### Characterization of the TME in different subtypes

**Figure 4:**
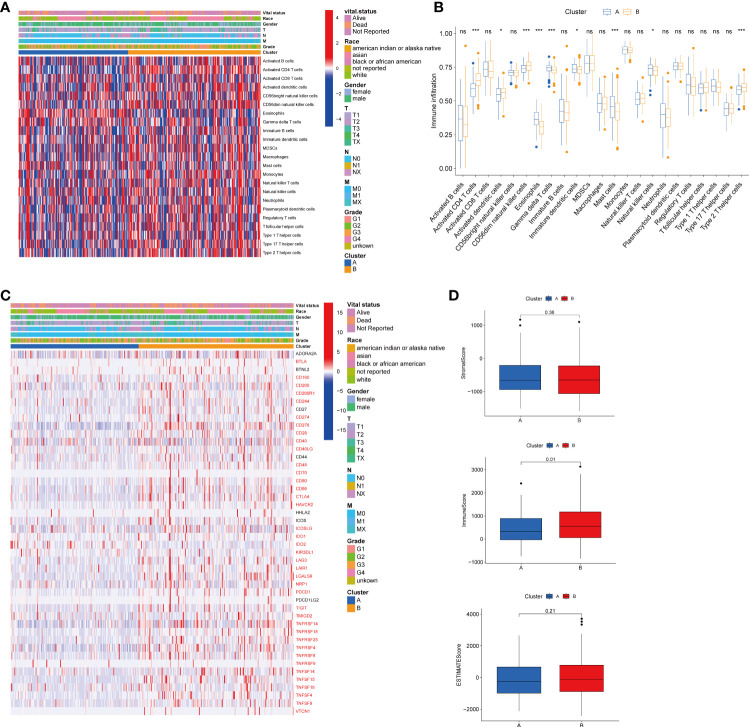
Correlations of tumor immune cell microenvironments and two HCC subtypes. **(A)** Heatmap of the tumor-infiltrating cells and clinical features in two HCC subtypes. **(B)** Expression abundance of 23 infiltrating immune cell types in the two HCC subtypes. **(C)** Immune checkpoints heatmap between the two subtypes, the red mark representing the checkpoints that are differentially expressed, with p < 0.05. **(D)** Correlations between the TME score and the two HCC subtypes. HCC, hepatocellular carcinoma; TME, tumor microenvironment. *p < 0.05, ***p < 0.001, ns, no significant difference.

To explore the role of TRGs in HCC TME, we evaluated the associations between the two subtypes and 23 human immune cell subsets using the ssGSEA method. We observed significant variations in the infiltration of some immune cells between the two subtypes (**Figures 4A–B**). The infiltration level of eosinophils, gamma delta T cells, immature dendritic cells, mast cells, and natural killer cells were remarkably elevated in subtype A, while activated CD4 T cells, activated dendritic cells, CD56dim natural killer cells, and type 2 T helper cells were significantly overexpressed in subtype B. We further investigated the profile of immune checkpoints between the two subgroups (**Figure 4C**). We discovered that most immune checkpoints were differentially expressed between the two groups, including *CTLA4*, *LAG3*, *PDCD1* (PD-1), and *CD274* (PD-L1), suggesting a potential role of the tyrosine metabolism-related subtypes in immunotherapy. Besides, by using the ESTIMATE R package, we evaluated the TME score, which included stromal score, ESTIMATE score, and immune score, between the two subtypes. For the TME score, the stromal or immune scores represented the content of stromal or immune cells in the TME, and the ESTIMATE scores implied aggregation of immune or stromal scores in the TME. We only found a higher immune score in subtype A, while there was no statistical difference in stromal score and ESTIMATE score between the two subtypes (**Figure 4D**).

### Construction and validation of TRG risk model

**Figure 5:**
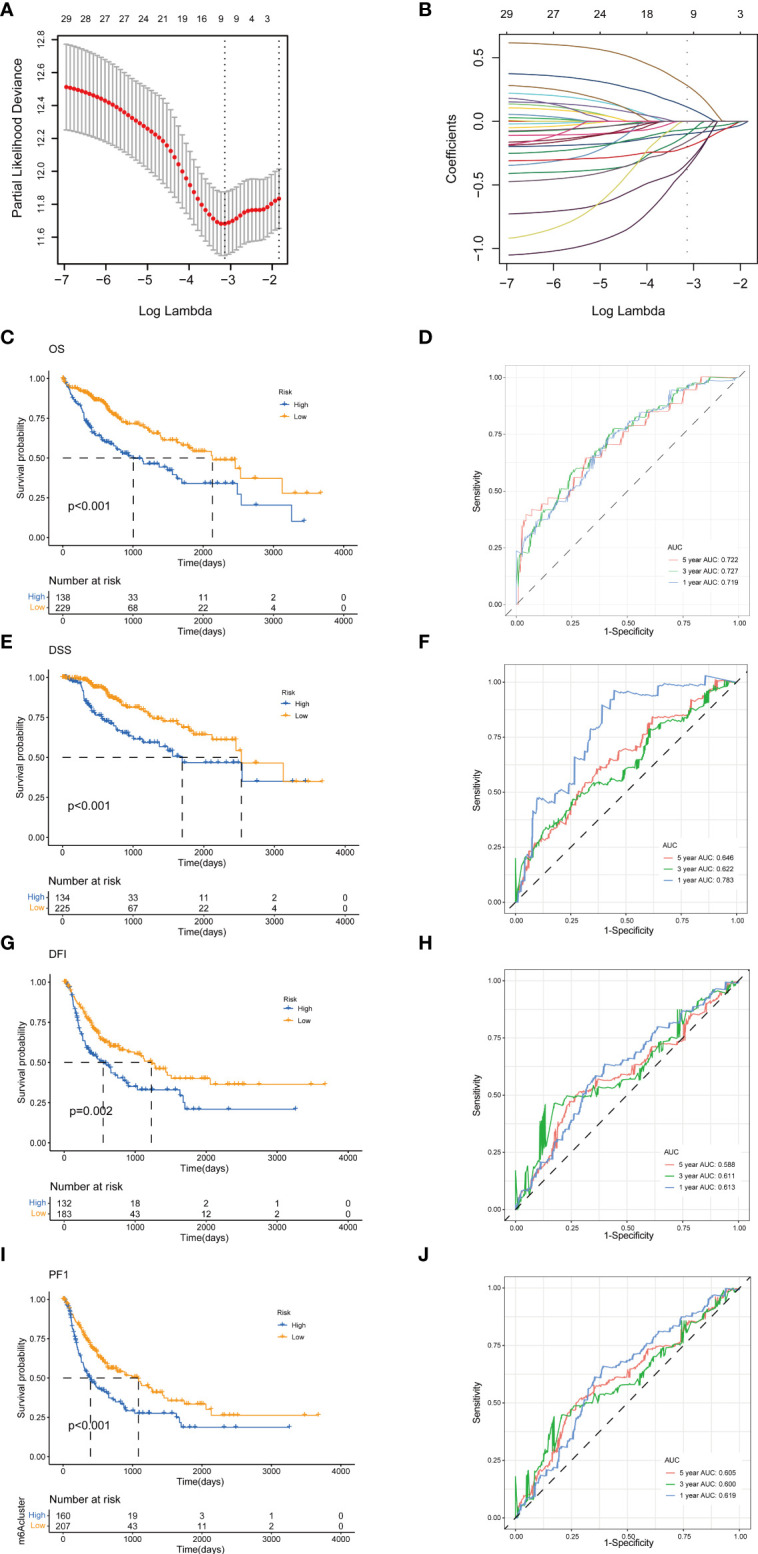
Construction of tyrosine metabolism-related genes prognostic model in the training set. **(A)** Ten-time cross-validation for tuning parameter selection by LASSO regression. **(B)** The screening of coefficients under LASSO analysis. A vertical line is drawn at the value chosen by 10‐fold cross‐validation of overall survival. Kaplan-Meier curves for survival outcomes of the two risk subtypes according to the OS **(C)**, DSS **(E)**, DFI **(G)**, and PFI **(I)** (log-rank tests, p< 0.01). ROC curves to predict the sensitivity and specificity of 1-, 3-, and 5-year survival rates according to the risk score based on the OS **(D)**, DSS **(F)**, DFI **(H)**, and PFI **(J)**. LASSO, least absolute shrinkage and selection operator; OS, overall survival; DSS, disease-specific survival; DFI, disease-free interval; PFI, progression-free interval; ROC, receiver operating characteristic.

To establish a predictive prognostic model for HCC patients, we explored the prognostic genes in the HCC TCGA training set. By using the univariate Cox regression analysis, HCC patients were classified into high- and low-risk groups based on the optimal cutoff of each gene. We identified that nine TRGs were associated with an inferior OS, and twelve genes were related to a favorable OS in HCC patients (**Supplementary Figure 1A**). Meanwhile, similar gene risk distribution was discovered in the DSS, DFI and PFI (**Supplementary Figure 1B–D**). Then, we performed LASSO and multivariate COX analysis on the prognostic-related genes with 10-fold cross-validation to narrow the gene scope (**Figures 5A, B**). Subsequently, we obtained a five-gene signature model with two high-risk genes (*METTL6* and *LCMT1*), and three low-risk genes (*GSTZ1*, *ADH4*, and *ADH1A*). The risk score of each HCC patient was calculated according to the following formula:

Risk score = (0.24* expression of *METTL6*) + (0.22* expression of *LCMT1*) + (−0.16* expression of *GSTZ1*) + (−0.23* expression of *ADH4*), + (−0.18* expression of *ADH1A*).

The HCC patients were categorized into high-risk and low-risk groups based on the best cutoff of the risk score. The Kaplan-Meier analysis revealed that the patients in high-risk group had an unfavorable OS (log-rank test, p<.001; **Figure 5C**), DSS (log-rank test, p<.001; **Figure 5E**), DFI (log-rank test, p = .002; **Figure 5G**), and PFI (log-rank test, p<.001; **Figure 5I**). We further performed the time-dependent receiver operating characteristic (ROC) curve with the area under the curve (AUC). The AUC values of 1-, 3-, and 5-year survival rates of OS-related prognostic subgroups were 0.719, 0.727, and 0.722, respectively (**Figure 5D**). We also analyzed the AUC values of 1-, 3-, 5-year survival rates of DSS (0.783, 0.622, and 0.644, respectively; **Figure 5F**), DFI (0.613, 0.611, and 0.588, respectively; **Figure 5H**), and PFI (0.619, 0.600, and 0.605, respectively; **Figure 5J**).

**Figure 6:**
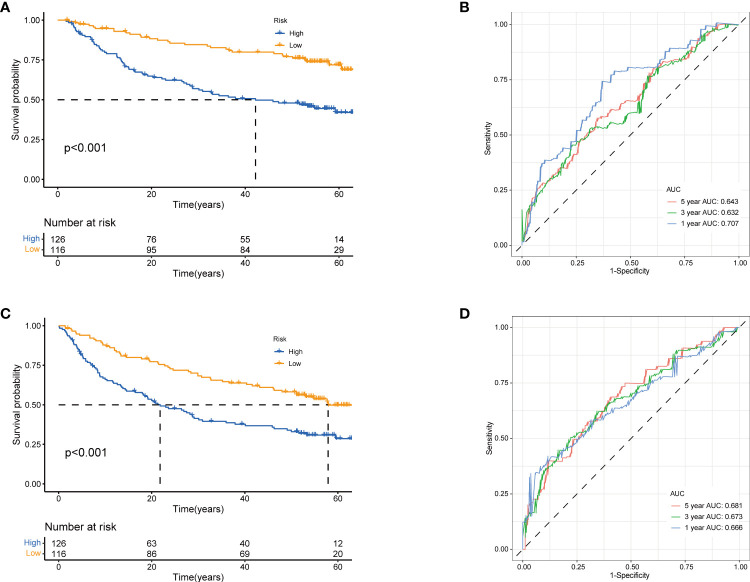
Validation of prognostic model based on TRGs. Kaplan-Meier survival curves of high- and low-risk groups in validation dataset GSE14520 **(A)**, OS. **(C)**, RFS (log-rank tests, p<.001). The receiver operating characteristic curve for predicting 1-year, 3-year, and 5-year OS **(B)** and RFS **(D)** of HCC patients in GSE14520. TRGs, tyrosine metabolism-related genes; HCC, hepatocellular carcinoma; RFS, relapse-free survival.

**Figure 7:**
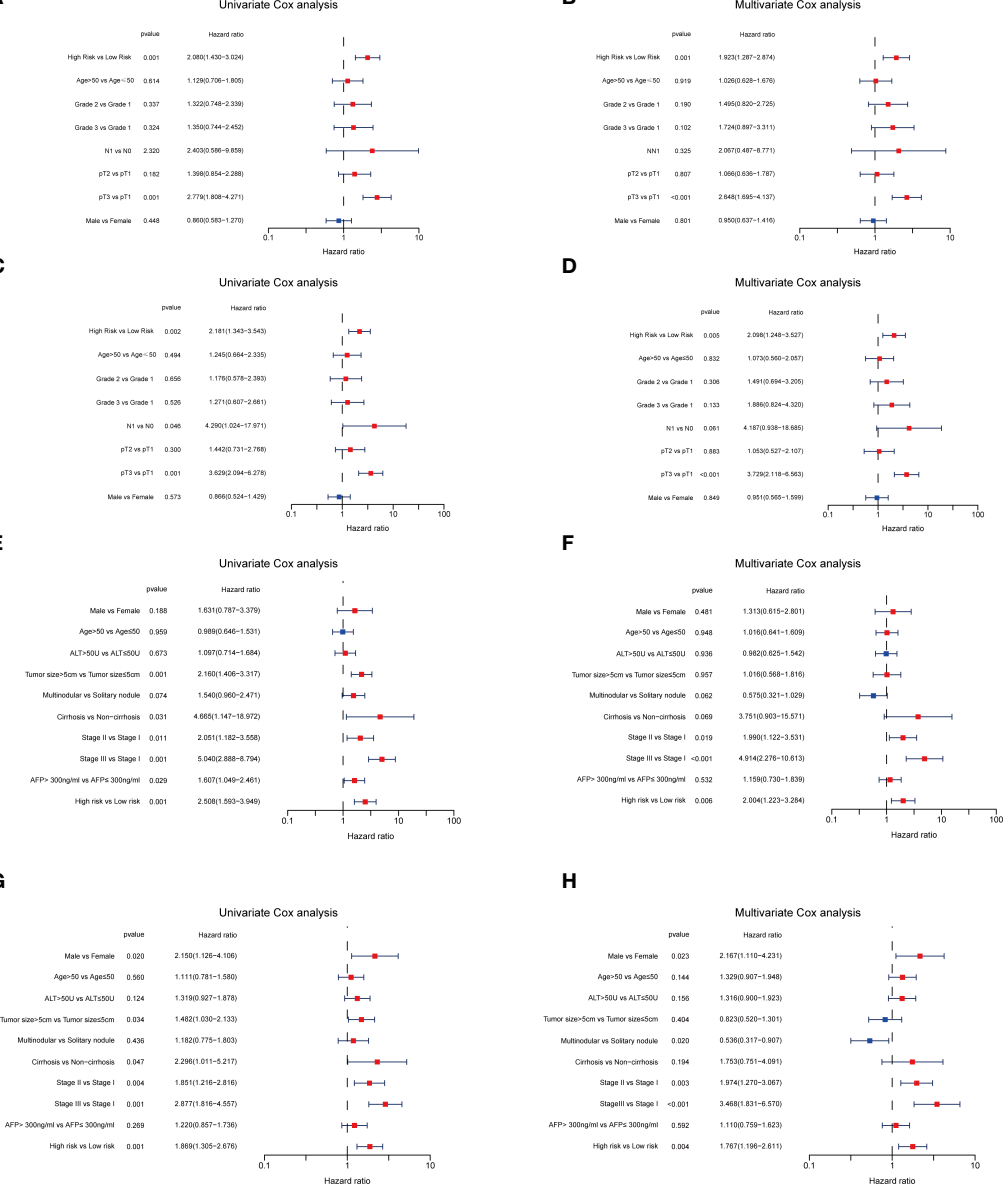
Independent prognosis analyses of TRGs risk model in TCGA and GES14520 HCC cohorts. **(A, B)**, Univariate and Multivariate Cox regression of risk score based on OS in TCGA HCC cohort. **(C, D)**, Univariate and Multivariate Cox regression of risk score based on DSS in TCGA HCC cohort. **(E, F)**, Univariate and Multivariate Cox regression of risk score based on OS in GSE14520 HCC cohort. **(G, H)**, Univariate and Multivariate Cox regression of risk score based on RFS in GSE14520 HCC cohort. TRGs, tyrosine metabolism-related genes; HCC, hepatocellular carcinoma; TCGA, The Cancer Genome Atlas; OS, overall survival; DSS, disease-specific survival; RFS, relapse-free survival.

Next, to validate the prognostic performance of the TRGs model, we calculated the efficiency in the validation set (GSE14520). Similarly, we gained the parallel results in the validation sets, indicating the prognostic model of TRGs had an excellent predictive prognostic accuracy for HCC patients (**Figures 6A–D**). To further explore the predictive role of tyrosine metabolism-related signature, we performed univariate and multivariate Cox proportional hazards regression analysis in the HCC TCGA and GSE14520 datasets. The results showcased that the tyrosine metabolism-related signature was an independent risk factor for OS and RFS in patients with HCC (**Figures 7A–H**).

In addition, we further compared our signature with two other metabolism-related prognostic models (28, 29). In Wu et al.’s study (28), they established a six metabolism-related mRNAs prognostic model for HCC patients. The ROC curve of AUC values of 1-, 3-, and 5-year survival rates of OS-related prognostic subgroups were 0.583, 0.595, and 0.582, respectively (**Supplementary Figure 2A**), and the AUC values of 1-, 3-, 5-year survival rates of RFS were 0.599, 0.568, and 0.528, respectively (**Supplementary Figure 2B**). For Dai et al.’s study (29), we also analyzed its predictive ability in HCC. The ROC curve of AUC values of 1-, 3-, and 5-year survival rates of OS-related prognostic subgroups were 0.603, 0.617, and 0.631, respectively (**Supplementary Figure 2C**), and the AUC values of 1-, 3-, 5-year survival rates of RFS were 0.616, 0.623, and 0.601, respectively (**Supplementary Figure 2D**). Taken together, our TRGs signature presented a better performance in predicting the prognosis of HCC patients.

### Evaluation of TME and immune checkpoints in TRG risk models

**Figure 8:**
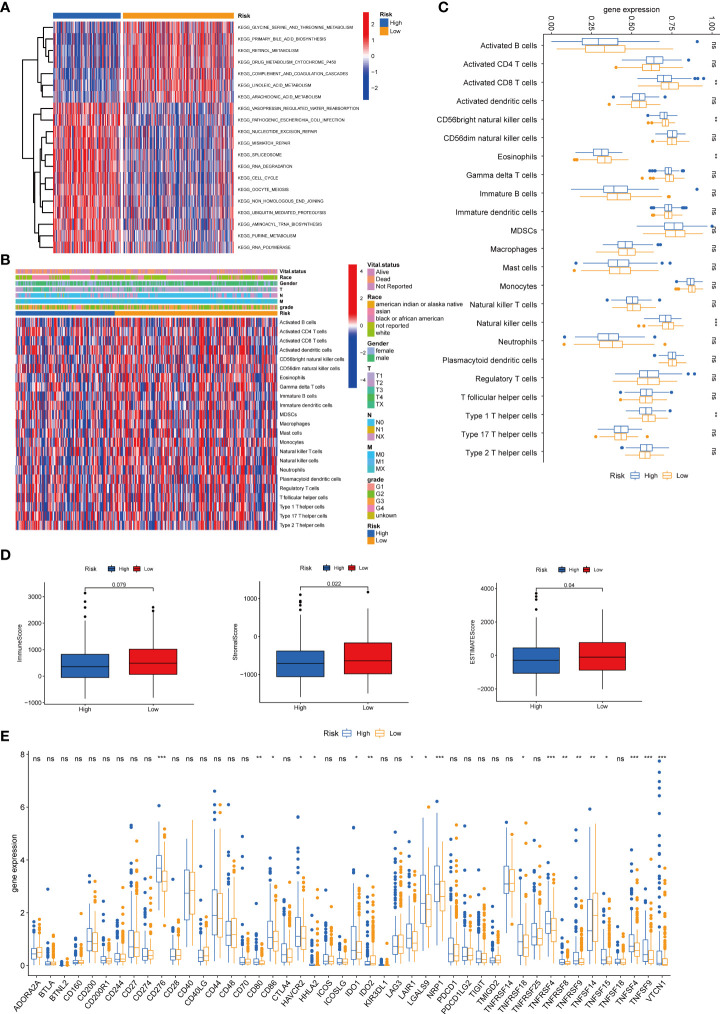
Correlations of tumor immune cell microenvironments and two TRGs prognostic subtypes. **(A)** GSVA of biological pathways between two risk groups, in which red represent activated and blue inhibited pathways, respectively. **(B)** Heatmap of the clinicopathologic characteristics and tumor-infiltrating cells in the two risk groups. **(C)** Expression abundance of 23 infiltrating immune cell types in the two risk subtypes. **(D)** Correlations between the TME score and the two risk subtypes. **(E)** Expression of immune checkpoints between the two risk subtypes. *p < 0.05, **p < 0.01, ***p < 0.001, ns, no significant difference. TRGs, tyrosine metabolism-related genes; GSVA, gene set variation analysis; TME, tumor microenvironment.

We then performed the GSVA enrichment analysis between the two groups and found distinct functional enrichment in the two subtypes. The high-risk group was enriched in pathways of nucleotide excision repair, cell cycle, mismatch repair, and spliceosome, while the low-risk group was positively correlated with drug metabolism cytochrome p450 and complement and coagulation cascades (**Figure 8A**). To further explore the relationship between the tyrosine metabolism-related risk model and the TME signature, we investigated the correlation of risk subtypes and immune infiltration cells of HCC by the ESTIMATE algorithm (**Figures 8B–C**). The infiltration level of activated CD8 T cells, CD56bright natural killer cells, eosinophils, natural killer cells, and type 1 T helper cells were obviously elevated in the low-risk group. Besides, we calculated the TME score of high and low-risk groups and revealed that the stromal score and ESTIMATE score were upgraded in the low-risk subtype (**Figure 8D**). We then investigated the profile of immune checkpoints between the two subtypes, and we revealed that amounts of immune checkpoints were distinctively expressed between the two groups, such as *CD276*, *CD80*, *CD86*, and *HAVCR2* (**Figure 8E**).

### Analysis and validation of the five TRGs for the prognostic signature

**Figure 9:**
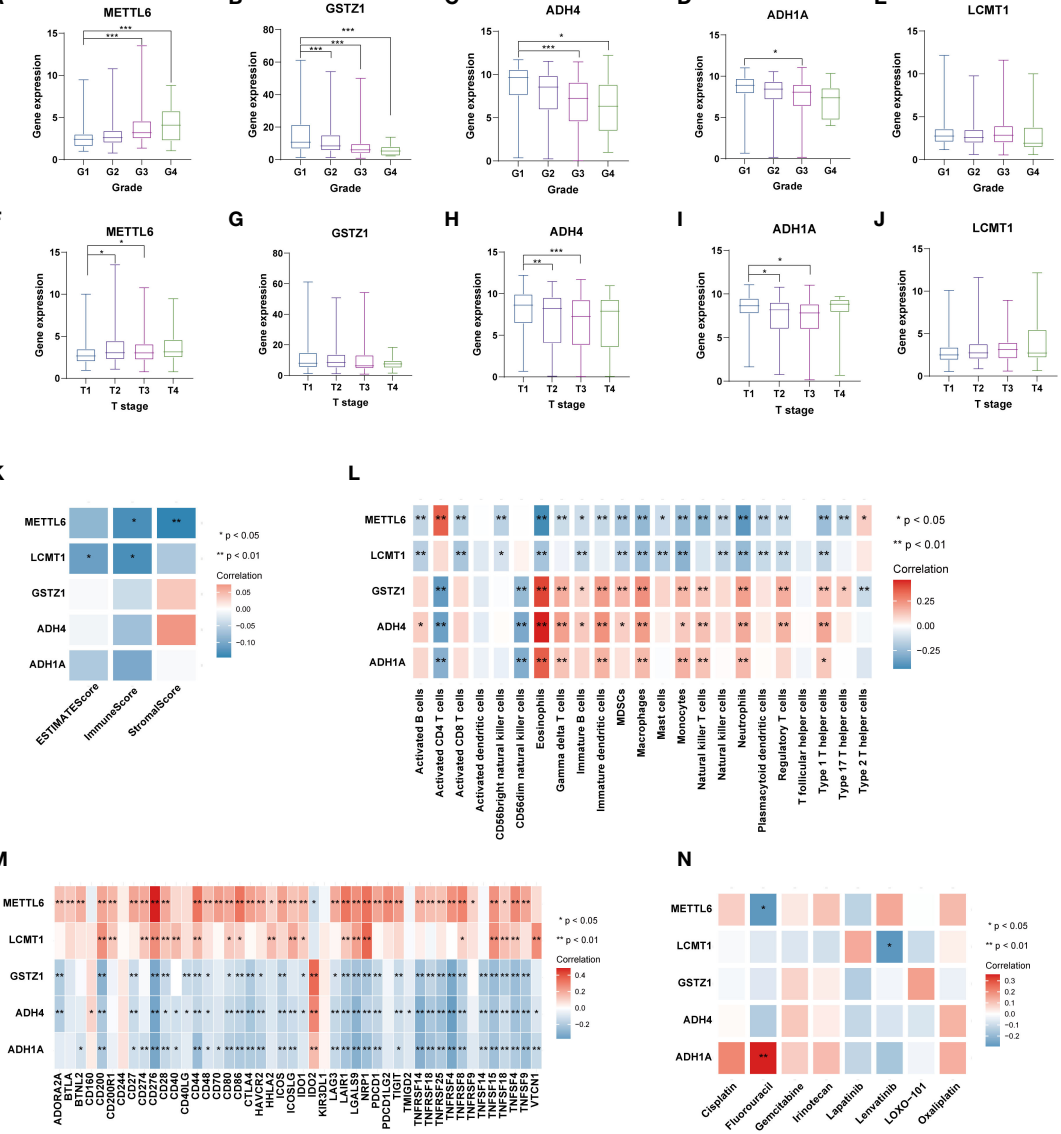
Analysis of five TRGs for the prognostic signature, and their correlations of tumor immune infiltrating cells and therapeutic drugs. The boxplot showing the relationship of METTL6 **(A)**, GSTZ1 **(B)**, ADH4 **(C)**, ADH1A **(D)**, and LCMT1 **(E)** expression and grade stratification. The boxplot depicting the correlation of METTL6 **(F)**, GSTZ1 **(G)**, ADH4 **(H)**, ADH1A **(I)**, and LCMT1 **(J)** expression and T stage. **(K)** The correlation of five TRGs and TME score. **(L)** The relationship between five TRGs and 23 activated immune cells. **(M)** The correlation of five TRGs and immune checkpoints. **(N)** The relationship between five TRGs and common therapeutic drugs for HCC. *p<0.05, **p< 0.01, ***p < 0.001. TRGs, tyrosine metabolism-related genes; TME, tumor microenvironment.

We further analyzed the expression levels of five prognostic TRGs in HCC patients. HCC is a complex disease driven by various extrinsic and intrinsic factors; we further investigated the TRGs risk model with other clinical signatures, such as tumor grade, stage, AFP level, cirrhosis and HBV status, and were summarized in **Tables 1**, **2**. We explored that the expression of *METTL6*, *GSTZ1*, *ADH4*, and *ADH1A* were related to the grade classification (**Figures 9A–E**), and *METTL6*, *ADH4*, and *ADH1A* were also associated with the T stage of tumors (**Figures 9F–J**). We also found that the TRGs-related signature was associated with tumor size, HBV status, AFP level, ALT level, and tumor grade. Furthermore, we further compared our prognostic signature with multi-platform studies from the TCGA. We discovered that the high-risk group was associated with the subclass of MS1 in Shimada’s classification (30), G1/2 and G3 in Boyault’s subtype (31), and Proliferation in Chiang’s classification (32) (**Supplementary Table 2**). All the subclasses have been documented as the “proliferative” and “aggressive” phenotypes and characterized by inferior patient prognosis, which was in line with our study.

**Table 1:** Clinicopathologic characteristics of TCGA HCC patients according to the Tyrosine -related signature.

Variables	Risk		*P* value
	High risk	Low risk	
**Gender**			0.007
Female	56	60	
Male	81	163	
**Age at diagnosis (years)**			0.893
≤ 50	29	45	
> 50	108	178	
**T stage**			0.001
T1	49	128	
T2	42	48	
T3	40	40	
T4	6	7	
**N stage**			0.036
N0	99	150	
N1	3	0	
NX	35	73	
**M stage**			0.158
M0	103	158	
M1	3	1	
MX	31	64	
**Grade**			0.035
I	16	38	
II	66	109	
III	46	73	
IV	9	3	
**HBV history**			0.007
No	56	59	
Yes	13	16	
Unknown	68	148	
**HCV history**			0.165
No	101	146	
Yes	18	49	
Unknown	18	28	
**Cirrhosis**			0.629
No	119	186	
Yes	16	31	

**Table 2:** Clinicopathologic characteristics of GSE14520 HCC patients according to the Tyrosine -related signature.

Variables	Risk		*P* value
	High risk	Low risk	
**Gender**			0.848
Female	17	14	
Male	109	102	
**Age at diagnosis (years)**			0.021
≤ 50	72	55	
> 50	55	63	
**ALT**			0.695
>50U/L	54	46	
≤50U/L	72	70	
**AFP**			<0.001
>300ng/ml	77	33	
≤300ng/ml	47	81	
**Tumor Size**			0.001
>5cm	59	29	
≤5cm	67	86	
**Multinodular**			0.001
No	88	102	
Yes	38	14	
**T stage**			<0.001
T1	34	62	
T2	42	36	
T3	39	12	
**Cirrhosis**			0.959
No	10	9	
Yes	116	107	

Next, we then assessed the correlations between the TME scores and the five TRGs, and it is found that *METTL6* and *LCMT1* were negatively correlated with the TME scores (**Figure 9K**). We further investigated the relationship between the five TRGs and immune infiltrating cells. We observed that the *METTL6* and *LCMT1* were negatively related to most immune cells, whereas *GSTZ1*, *ADH4*, and *ADH1A* were positively correlated with various immune cells, except for activated CD4 T cell and CD56dim natural killer cell (**Figure 9L**). Besides, we also explored the correlations of five TRGs and immune checkpoints. It is obvious that *METTL6* and *LCMT1* were positively associated with a large number of immune checkpoints, while the *GSTZ1*, *ADH4*, and *ADH1A* were negatively related to a great proportion of immune checkpoints, except for *IDO2* and *CD160* (**Figure 9M**). In addition, we also investigated the correlations of five TRGs with the sensitivity of common chemotherapeutic drugs and targeted therapeutic drugs of HCC (**Figure 9N**). We discovered that, unlike *METTL6*, *ADH1A* was positively related to the response of Fluorouracil, and *LCMT1* was negatively correlated to the therapeutic effect of Lenvatinib, indicating that these prognostic-related TRGs may also influence the therapeutic efficacy of HCC.

**Figure 10:**
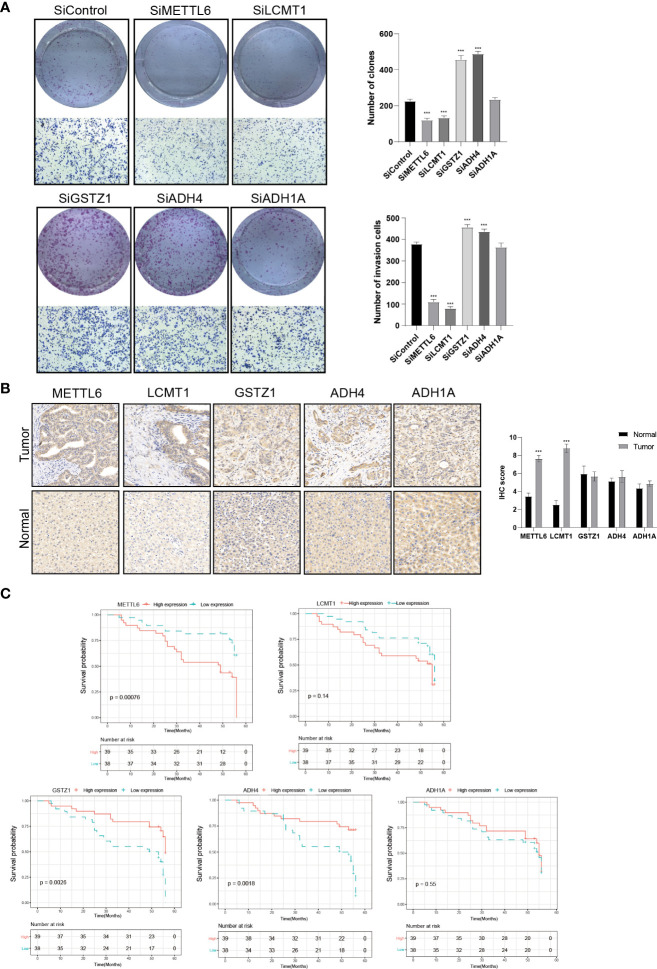
Validation of the Five prognostic TRGs by functional analysis. **(A)** Clone formation and invasion analysis of Hep3B cells depleted with the five TRGs. **(B)** IHC analysis of the five TRGs in the Xiangya HCC cohort (n = 97), including normal liver tissue (n = 20) and HCC tumor tissues (n = 77). **(C)** Kaplan-Meier curve analysis of the five TRGs in HCC patients. Patients were divided into high- and low-expression groups based on the median expression of each gene. TRGs, tyrosine metabolism-related genes; HCC, hepatocellular carcinoma; IHC, immunohistochemistry. ***p < 0.001.

To further validate our exploration, we first performed clone formation, transwell invasion, and IHC analysis to validate our results. We silenced the expression of five TRGs through siRNA to further verify the biological functions of *METTL6, GSTZ1, ADH4, ADH1A*, and *LCMT1*. The results of colony formation indicated that depletion of *METTL6*, and *LCMT1* inhibited the colony formation ability of Hep3B cells, while *GSTZ1* and *ADH4* accelerated the colony formation ability of the Hep3B cells, which was in line with their role in the prognostic model. However, there was no statistical significance upon *ADH1A* depletion and it need further exploration. Consistently, the silence of *METTL6* and *LCMT1* decreased cancer cell migration, and depletion of *GSTZ1* and *ADH4* increased the cancer cell migration ability (**Figure 10A**). In addition, we performed the IHC analysis of the five TRGs in the Xiangya HCC cohort, which included 77 HCC tumor tissues and 20 normal liver tissues. The IHC analysis revealed that METTL6 and LCMT1 were highly expressed in liver tumor tissues, while we did not observe significant differences in the GSTZ1, ADH1A and ADH4 expression between HCC tumors and normal tissues (**Figure 10B**). The survival analysis indicated that the overexpression of METTL6 was associated with an inferior prognosis, while the high expression of GSTZ1 and ADH4 had a favorable prognosis, which was consistent with their prognostic role in mRNA level (**Figure 10C**).

In addition, we then explored the expression of five TRGs in HCC cell lines by using the Cancer Cell Line Encyclopedia (CCLE) database, which contained 25 different HCC cell lines. The expression of five TRGs in diverse HCC cell lines were summarized in **Supplementary Table 3** and **Supplementary Figure 3**. We discovered that SNU-423, SNU-182, SNU-761, and Huh-1, JHH5, SNU-878 cell lines represented the high-risk and low-risk subtypes, respectively. We further evaluated the responsiveness of these cell lines to the common HCC chemotherapeutic and targeted drugs through the Genomics of Drug Sensitivity in Cancer (GDSC) platform, which included 16 different cell lines (**Supplementary Table 4**). We only detected the responsiveness of SNU-423 and Huh-1 cell lines in the GDSC database. The results demonstrated that the SNU-423 was more sensitive to the 5-Fluorouracil, Oxaliplatin, and Irinotecan (**Supplementary Figures 4A-C**), indicating a higher degree of malignancy of the tumor and was consistent with the model classification of high-risk. Meanwhile, we also found that Huh-1 was more sensitive to Sorafenib compared with SNU-423 (**Supplementary Figure 4D**), which provided new perspectives for individualized therapy.

Moreover, we further analyzed the protein expression of the five TRGs using the Clinical Proteomic Tumor Analysis Consortium (CPTAC) cohort. We only detected four of the five TRGs in the proteomics data. We observed that ADH4, GSTZ1, and ADH1A were downregulated in HCC tumor tissues, while the LCMT1 was overexpressed in HCC tumor tissues compared with the normal tissues (**Supplementary Figure 5**), which were consistent with the mRNA expression level of HCC. Besides, we confirmed the protein expression of the five genes in human tissue samples. As illustrated in **Supplementary Figure 6**, ADH4, GSTZ1, and ADH1A were downregulated in HCC tumor tissues compared with the normal tissues, which were consistent with their role in HCC. However, we did not observe significant differences in the METTL6 and LCMT1 expression between HCC tumors and normal tissues, which needs further exploration.

## Discussion

Tyrosine metabolism plays a vital role in substance catabolism, the disturbance of which has been recognized as the hallmark of disease development and tumorigenesis (33, 34). Recent studies suggested that tyrosine metabolism may have a potential relationship with the tumor microenvironment and immunogenicity of tumors (17, 35). However, the overall effect and the relationship between tyrosine metabolism gene signatures and tumor immune infiltration mediated by multiple TRGs in HCC have not been reported. In the meantime, it is crucial to investigate the TRGs molecular features for a better comprehension of the biological relationship between clinical outcomes and tumor immune environment.

In the present study, we revealed a landscape of tyrosine metabolism-related genes and TME signatures in HCC. The results of the present study demonstrated genetic and transcriptional variations in RRGs in HCC. We revealed two distinct molecular subtypes based on the expression of 42 tyrosine metabolism-related genes. Compared to patients in Cluster B, patients in Cluster A had a superior survival time. The GSVA enrichment analysis revealed that Cluster A was significantly enriched in various substances’ metabolism, such as tyrosine metabolism, fatty acid metabolism, and histidine metabolism, etc., and it can be considered highly metabolic. The pathways enriched in Cluster A indicated that they played essential roles in the development of HCC and there was crosstalk among the tyrosine metabolism and other signaling pathways. We also found features of the TME between the two clusters, which were characterized by some activation pathways’’ activation, such as cancer-related pathways, various virus infections and substance metabolism. Besides, we established an effective prognostic TRGs risk model that included five TRGs (*METTL6*, *LCMT1, GSTZ1*, *ADH4*, and *ADH1A*) and validated its predictive ability. The patients from the two subtypes had remarkably diverse survival outcomes. The multivariate Cox regression model proved that TRGs signature was an independent risk factor adjusting for clinical characteristics such as tumor size, nodule number, cirrhosis status, and alpha-fetoprotein (AFP) level. In addition, we explored the relationship between the TRGs risk model and TME, and revealed the strong association of *METTL6*, *LCMT1, GSTZ1*, *ADH4*, and *ADH1A* with the tumor immune infiltration and immune checkpoints. Unlike the previous work for individually analyzing the expression pattern and prognostic value of five particular genes involved in tyrosine catabolism in HCC (36), the current study demonstrated a comprehensive analysis of TRGs and the immune microenvironment. To the best of our knowledge, this is the first study which presents an unbiased tyrosine metabolism-related risk model in HCC. The TRGs prognostic model can be applied to categorize the prognosis of HCC patients, which will help to better understand the molecular mechanism of HCC and provide new insights for targeted and immune therapy.

Recently, there are an increasing number of studies working on the prognostic model of HCC based on ferroptosis phenotypes (37), cuproptosis-related genes (38), m^5^C regulatory genes (39), hypoxia-related angiogenic genes (40), et al. They all showed meaningful clinical implications to some extent. As we know the liver is the primary organ that participates in tyrosine catabolism. It has been reported that the disturbance of tyrosine metabolism could cause a variety of diseases, and patients suffering from hereditary tyrosinemia are more likely to develop HCC (12). In HCC patients, the serum tyrosine is frequently upregulated, indicating an imbalance tyrosine metabolic process in HCC (13). Therefore, tyrosine catabolism signaling cascades play an irreplaceable role in the development and progression of HCC. By comparing our signature with two other metabolism-related prognostic models (28, 29), our TRGs signature presented a better performance in predicting the prognosis of HCC patients.

With greater numbers of tumor-infiltrating lymphocytes, tumor neoantigens, and checkpoints after standard therapy, the overall prognosis for HCC remains poor. Patients with HCC still exhibit heterogeneity in their outcomes despite recent advances in immunotherapy, suggesting the critical role of TME in the development and progression of HCC. Major biological components of TME include immune cells such as lymphocytes, granulocytes, and macrophages. These cells participate in various immunological reactions and activities, such as the inflammatory response orchestrated by tumors to aid in survival (41). TIICs, lymphocytes, fibroblasts, extracellular matrix (ECM), blood vessels, and inflammatory cells generated from bone marrow consist of the TME that surrounds tumor cells (42). Evidence has also demonstrated the critical influences of TME on tumor initiation, growth, and therapeutic resistance (43). In this study, we found that two TRGs subtypes differed considerably in the TME features as well as the relative abundance of 23 TIICs, which implies a vital role of TRGs in the development of HCC. In TRGs low-risk group, with a favorable prognosis, demonstrated an elevated infiltration level of activated CD8 T cells, CD56bright natural killer cells, eosinophils, natural killer cells, and type 1 T helper cells, implying that they play a beneficial role in HCC development. Besides, we discovered the diverse expression of immune checkpoints between the two TRGs subgroups, which helped to improve the efficacy of immunotherapy in the era of individualized therapy.

Among the five hallmark genes in the TRGs prognostic model, *METTL6* and *LCMT1* served as high-risk genes in the HCC cohort. *METTL6* is a transfer RNA methyltransferase, whose function and mechanism in cancer development are poorly understood. Michael et al. (44) found that *METTL6* was increased in highly proliferative luminal breast cancer, and Ignatova et al. (45) discovered that *METTL6* was a crucial regulator of HCC cell proliferation and that its absence reduced the pluripotency of murine stem cells. Our results were consistent with the previous study suggesting its indispensable role in tumor progression. *LCMT1* is a methyltransferase that catalyzes the methylation of protein phosphatase 2A(PP2A) (46). It has been reported that *LCMT1* was related to oxidative stress (47) and was overexpressed in neuroblastoma cells (48), while it has not been reported in HCC yet. Our study provides a new idea for the molecular function of HCC and can be further investigated. Besides, we also identified three favorable genes (*GSTZ1*, *ADH4*, and *ADH1A*) in the TRGs prognostic model. *GSTZ1* belongs to the Glutathione S-transferase (GST) superfamily and *GSTZ1* has been reported to be downregulated in HCC and performed as a tumor suppressor in the HCC progression (49, 50), which is in line with our findings. *ADH4* and *ADH1A* both belong to the alcohol dehydrogenase (ADH) superfamily and have revealed improved prognostic value in several cancer types, including non-small cell lung cancer, gastric cancer, and liver cancer (51–54). Our results were in accordance with previous studies, indicating its prognosis predictive role in HCC patients. In addition, we also revealed that *ADH1A* was positively related to the response of fluorouracil, and *LCMT1* was negatively correlated to the therapeutic effect of Lenvatinib, implying that these prognostic-related TRGs may also influence the therapeutic efficacy of HCC.

In the present study, we characterized the tyrosine metabolism-related genes signature in HCC and established a TRGs prognostic model based on five hallmark genes, showing a strong capacity in HCC prognosis prediction and immunogenicity evaluation. However, there are still some limitations in our study. Firstly, all the samples used in our investigation were collected retrospectively and analyses were performed on data from public databases. Hence, the inherent case selection bias may affect the results, and a more convincing prospective study is required to confirm our findings. Secondly, because of the finite sample size, large-scale cohort studies are essential for evaluating the value of this model. Thirdly, to improve the knowledge of tyrosine metabolism in the future, it is also vital to validate the molecular understanding based on *in vivo* and *in vitro* functional experiments. Finally, some crucial clinical information, including surgery, targeted therapy and chemoradiotherapy, was not available for analysis in the majority of datasets, which would have impacted on the prognosis of immune response and the tyrosine metabolism state.

## Conclusion

In summary, our integrative analysis demonstrated the possible molecular signature of TRGs in HCC and established a novel five-gene prognostic prediction model. The five hallmark genes (*METTL6*, *LCMT1*, *GSTZ1*, *ADH4*, and *ADH1A*) are prospective targets for determining the therapeutic efficacy of immunotherapy and targeted therapy, and accurately predict the survival of HCC patients. These findings highlight the significant clinical implications of TRGs and provide new perspectives for guiding individualized strategies for HCC patients.

## Data availability statement

The datasets presented in this study can be found in online repositories. The names of the repository/repositories and accession number(s) can be found in the article/**Supplementary Material**.

## Author contributions

JT contributed to the conception of the study. YZ, XL, GL and JT contributed to the data analysis, experiment implementation and figure generation. YZ wrote the manuscript. JT, YT and LZ revised the manuscript. All authors contributed to the article and approved the submitted version.

## Funding

This work was supported by the National Natural Science Foundation of China (82203160 & 82203353), the Hunan Provincial Natural Science Foundation of China (202JJ40815 & 202JJ40851 and the Youth Research Foundation of Xiangya Hospital, Central South University (2021Q16).

## Conflict of interest

The authors declare that the research was conducted in the absence of any commercial or financial relationships that could be construed as a potential conflict of interest.

## Publisher’s note

All claims expressed in this article are solely those of the authors and do not necessarily represent those of their affiliated organizations, or those of the publisher, the editors and the reviewers. Any product that may be evaluated in this article, or claim that may be made by its manufacturer, is not guaranteed or endorsed by the publisher.

## Glossary

**Table:**

AFP	alpha-fetoprotein
ADH	alcohol dehydrogenase
AUC	area under the curve
BP	biological process
CC	cellular component
CNVs	copy number variations
CDF	cumulative distribution function
DSS	disease-specific survival
DFI	disease-free interval
ECM	extracellular matrix
FPKM	fragments per kilobase million
GEO	Gene Expression Omnibus
GO	gene ontology
GSEA	Gene Set Enrichment Analysis
GSVA	gene set variation analysis
GST	Glutathione S-transferase
HCC	hepatocellular carcinoma
HPA	Human Protein Atlas
IC50	50% inhibiting concentration
KEGG	Kyoto Encyclopedia of Genes and Genomes
KM	Kaplan-Meier
LASSO	Least Absolute Shrinkage and Selection Operator Regression
MF	molecular function
OS	overall survival
PKU	phenylketonuria
PFI	progression-free interval
PCA	principal component analysis
PP2A	protein phosphatase 2A
ROC	receiver operating characteristic curve
RFS	relapse-free survival
ROC	receiver operating characteristic
ssGSEA	single sample gene set enrichment analysis
SNV	single nucleotide variation
TCGA	The Cancer Genome Atlas
TRGs	tyrosine metabolism-related genes
TME	tumor microenvironment
TIICs	tumor-infiltrating immune cells
WHO	World Health Organization.
