# Supplementary material for: Identification and validation of a tyrosine metabolism-related prognostic prediction model and characterization of the tumor microenvironment infiltration in hepatocellular carcinoma
Source: Front Immunol. 2022 Oct 20;13:994259. doi: 10.3389/fimmu.2022.994259 (PMC9633179; doi:10.3389/fimmu.2022.994259)
Supplement: Supplementary file 1 [file DataSheet_1.pdf]

Supplementary Materials for

Identification and Validation of a Tyrosine Metabolism-related Prognostic Prediction Model and Characterization of the Tumor Microenvironment Infiltration in Hepatocellular Carcinoma

Yangying Zhou[1,3], Xuanxuan Li[1,3], Guo Long[2,3], Yongguang Tao[4,5,6], Ledu Zhou[2,3], Jianing Tang[2,3*]

1. Department of Oncology, Xiangya Hospital, Central South University, Changsha, Hunan, 410008, China.
2. Department of Liver Surgery, Xiangya Hospital, Central South University, Changsha, Hunan, 410008, China.
3. National Clinical Research Center for Geriatric Disorders, Xiangya Hospital, Central South University, Changsha, Hunan, 410008, China.
4. Key Laboratory of Carcinogenesis and Cancer Invasion, Ministry of Education, Department of Pathology, Xiangya Hospital, Central South University, Changsha, Hunan, 410008, China.
5. Key Laboratory of Carcinogenesis of the Ministry of Health, Cancer Research Institute, School of Basic Medicine, Central South University, Changsha, Hunan, 410008, China.
6. Department of Thoracic Surgery, Hunan Key Laboratory of Tumor Models and Individualized Medicine, Second Xiangya Hospital, Central South University, Changsha, Hunan, 410008, China.

***Correspondence:** Jianing Tang, Department of Liver Surgery, Xiangya Hospital, Central South University, Changsha, 410008, Hunan, China. Email: tjn1995@whu.edu.cn

This PDF file includes:

Supplementary Figure 1. The overall predictive value of 42 TRGs in HCC patients.

Supplementary Figure 2. The ROC curves to predict the sensitivity and specificity of two other metabolism-related prognostic models.

Supplementary Figure 3. The gene expression of the five TRGs in HCC cell lines from the Cancer Cell Line Encyclopedia (CCLE) database.

Supplementary Figure 4. The drug sensitivity of HCC cell lines from the Genomics of Drug Sensitivity in Cancer (GDSC) platform.

Supplementary Figure 5. The protein expression of the four TRGs in HCC samples from the Clinical Proteomic Tumor Analysis Consortium (CPTAC) cohort.

Supplementary Figure 6. The protein expression of these five TRGs in HCC tissues.

Supplementary Table 1. Clinicopathologic characteristics of HCC patients.

Supplementary Table 2. Summary of correlation between the Tyrosine-related signature and other studies.

Supplementary Table 3. The gene expression of the five-tyrosine metabolism-related genes in HCC cell lines from the Cancer Cell Line Encyclopedia (CCLE) database.

Supplementary Table 4. The drug sensitivity of HCC cell lines from the Genomics of Drug Sensitivity in Cancer (GDSC) platform.

Supplementary Figure 1.

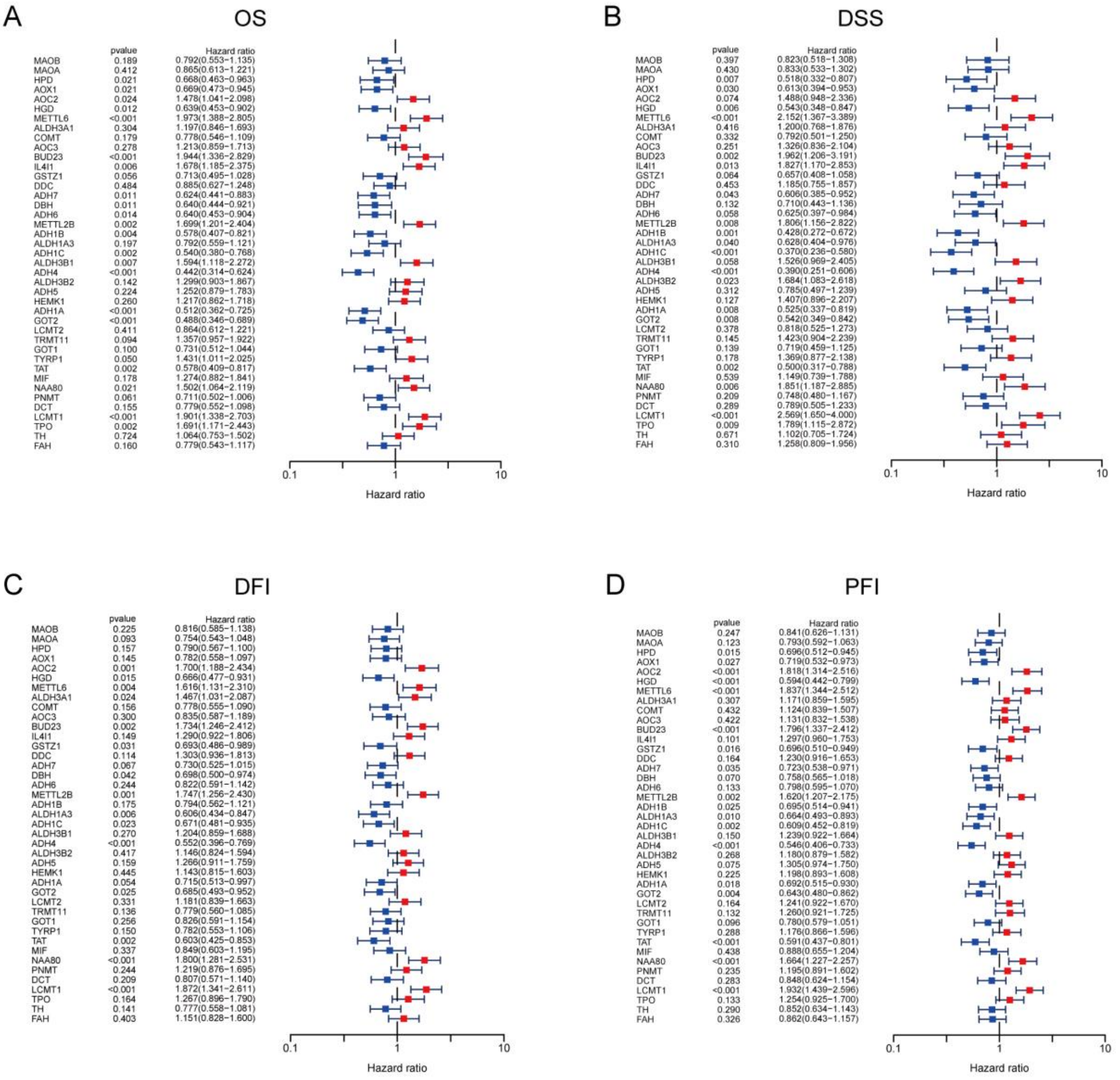

Supplementary Figure 1. The overall predictive value of 42 TRGs in HCC patients. Univariate Cox regression analysis of 42 TRGs associated with OS **(A)**, DSS **(B)**, DFI **(C)**, and PFI **(D)** in HCC patients. TRGs, tyrosine metabolism-related genes; HCC, hepatocellular carcinoma; OS, overall survival; DSS, disease-specific survival; DFI, disease-free interval; PFI, progression-free interval.

Supplementary Figure 2.

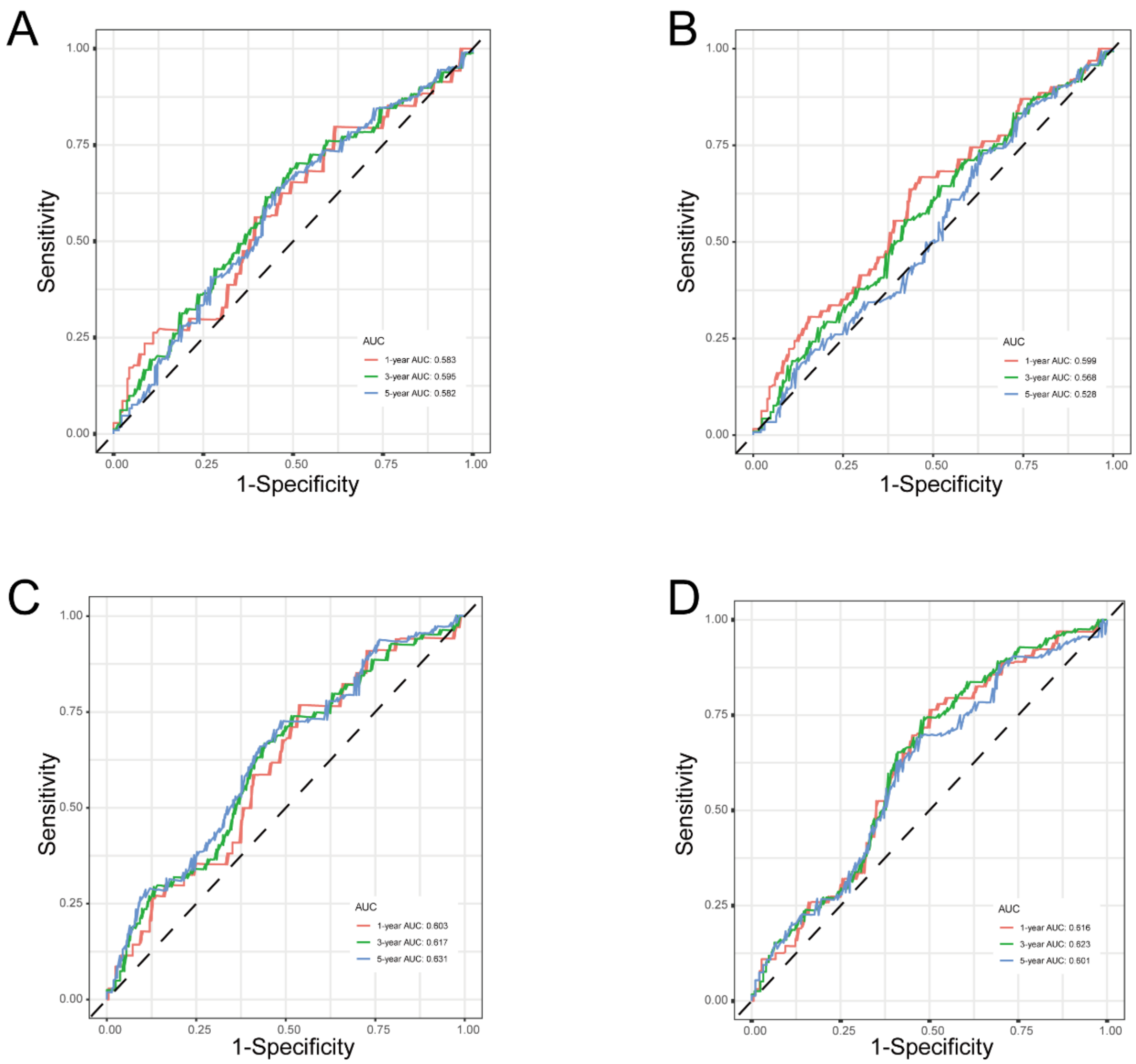

Supplementary Figure 2. The ROC curves to predict the sensitivity and specificity of two other metabolism-related prognostic models. (**A, B**) ROC curves to predict the sensitivity and specificity of 1-, 3-, 5-year OS (**A**) and RFS (**B**) according to the signature of Wu et al.'s study. (**C, D**) ROC curves to predict the sensitivity and specificity of 1-, 3-, 5-year OS (**C**) and RFS (**D**) according to the signature of Dai et al.'s study. ROC, receiver operating characteristic; OS, overall survival; RFS, relapse-free survival.

Supplementary Figure 3.

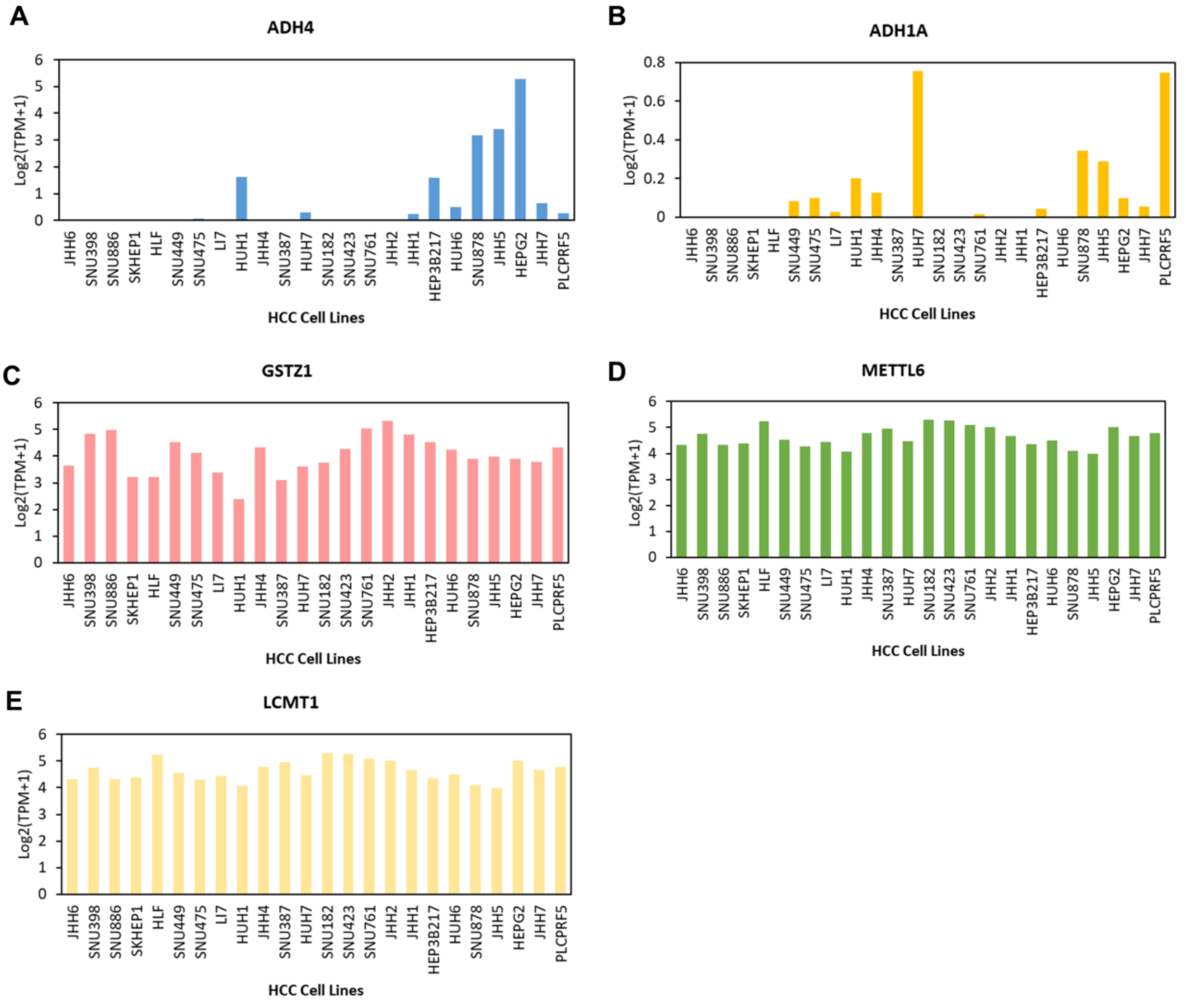

Supplementary Figure 3. The gene expression of the five TRGs in HCC cell lines from the Cancer Cell Line Encyclopedia (CCLE) database. The bar chart illustrates the expression level of *ADH4* (A), *ADH1A* (B), *GSTZ1* (C), *METTL6* **(D)** and *LCMT1* **(E)** in HCC cell lines. TRGs, tyrosine metabolism-related genes; HCC, hepatocellular carcinoma.

Supplementary Figure 4.

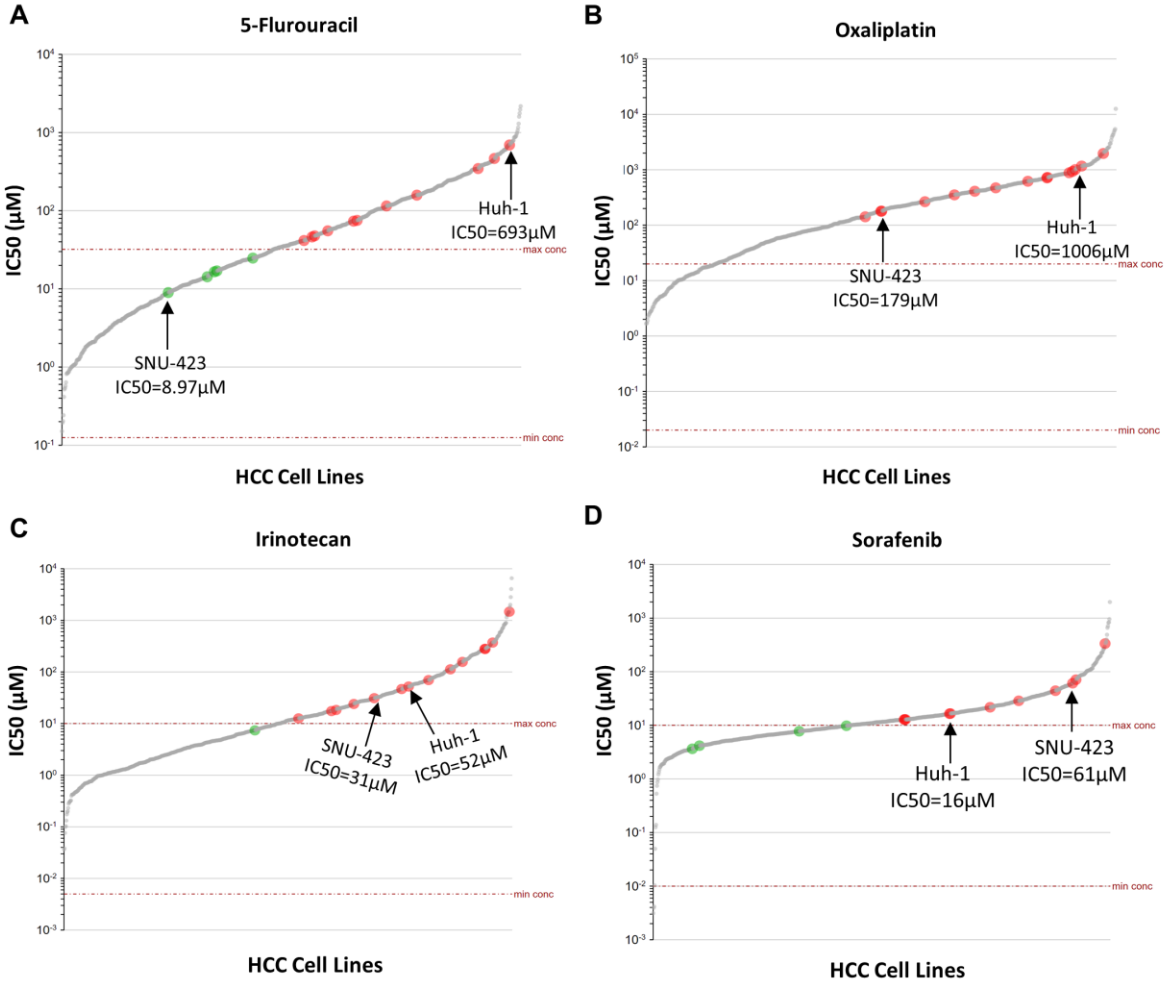

Supplementary Figure 4. The drug sensitivity of HCC cell lines from the Genomics of Drug Sensitivity in Cancer (GDSC) platform. The IC50 of different HCC cell lines responding to 5-Flurouracil (**A**), Oxaliplatin (**B**), Irinotecan (**C**), and Sorafenib (**D**). HCC, hepatocellular carcinoma; IC50, 50% inhibiting concentration.

Supplementary Figure 5.

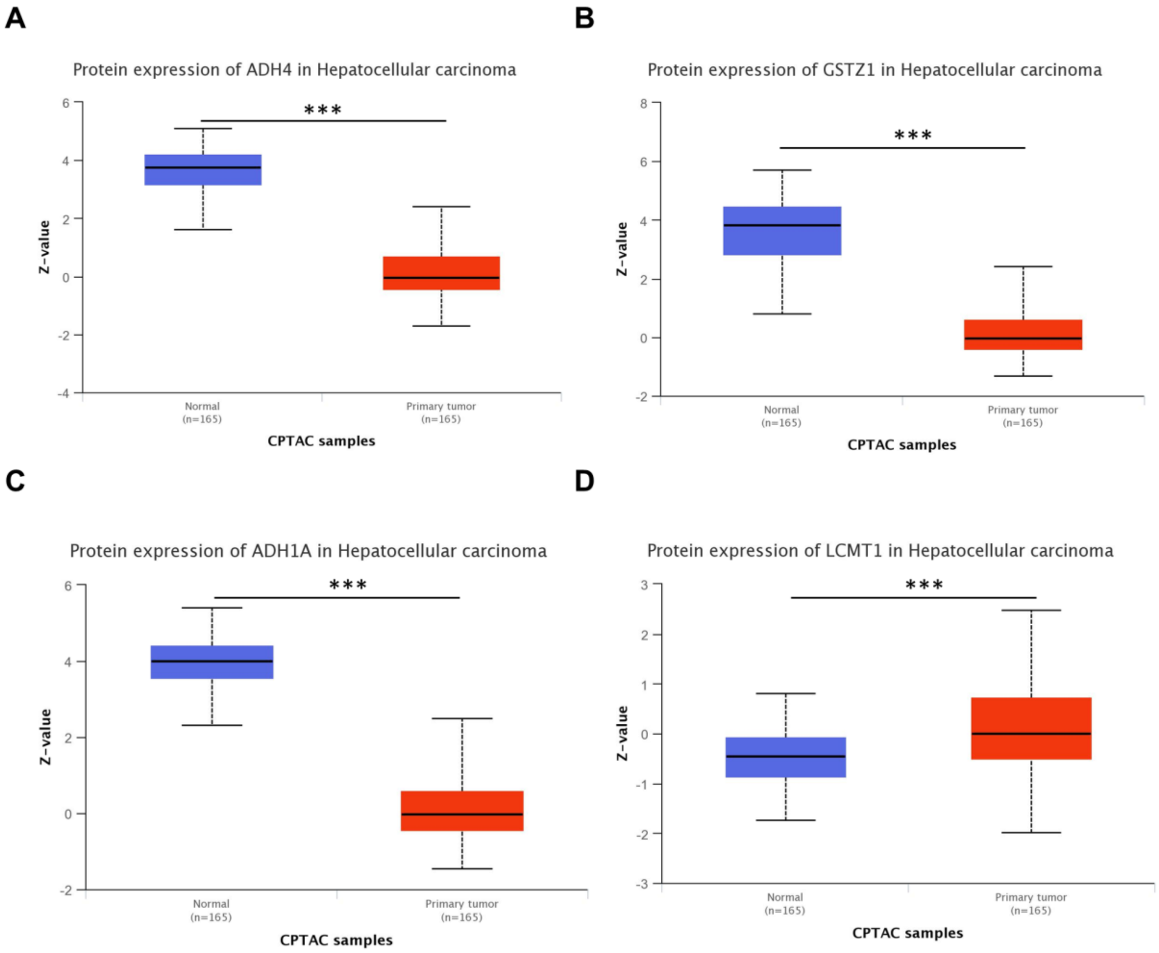

Supplementary Figure 5. The protein expression of the four TRGs in HCC samples from the Clinical Proteomic Tumor Analysis Consortium (CPTAC) cohort. The boxplot shows the proteomics level of ADH4 (**A**), GSTZ1 (**B**), ADH1A (**C**), and LCMT1 **(D)** in HCC normal and primary tumor tissues. ***$p < 0.001$.

Supplementary Figure 6.

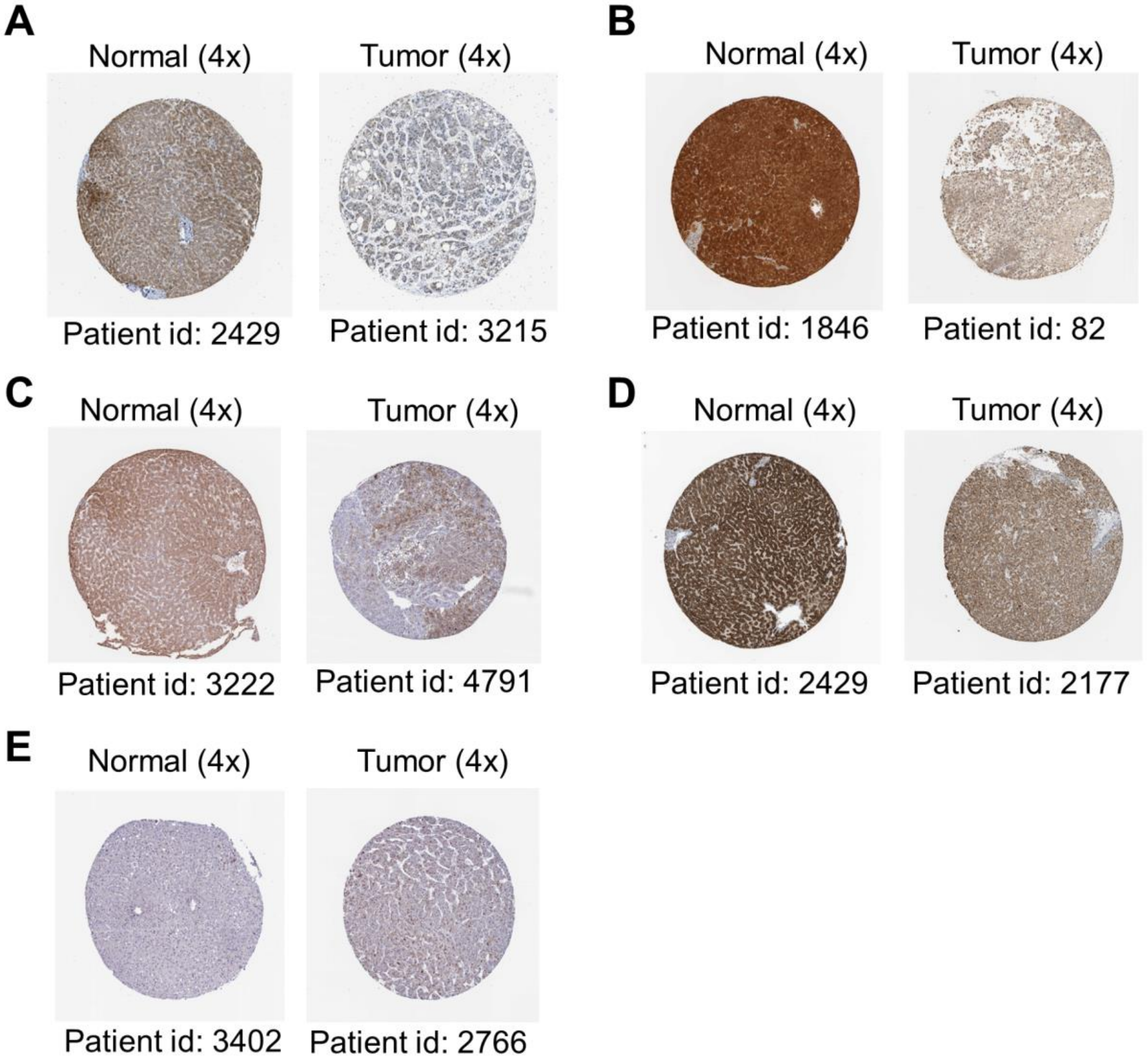

Supplementary Figure 6. The protein expression of these five TRGs in HCC tissues. Representative IHC images of the ADH4 **(A)**, GSTZ1 **(B)**, ADH1A **(C)**, METTL6 **(D)**, and LCMT1 **(E)** in HCC tumor and normal tissues. TRGs, tyrosine metabolism-related genes; HCC, hepatocellular carcinoma; IHC, immunohistochemistry.

Supplementary Table 1. Clinicopathologic characteristics of HCC patients.

Parameters	Numbers
**Age at diagnosis (years)**	
≤ 50	76
> 50	292
**Gender**	
Male	121
Female	250
**T**	
T1	181
T2	94
T3	80
T4	13
TX	3
**N**	
N0	252
N1	4
NX	115
**M**	
M0	266
M1	4
MX	101
**Grade**	
G1	55
G2	177
G3	122
G4	12
unknown	5
**Vital status**	
Alive	240
Dead	130
**Median follow-up (Months)**	19.4

Supplementary Table 2. Summary of correlation between the Tyrosine-related signature and other studies.

Classification	Risk		*P* value
	High risk	Low risk	
**Shimada's Classification**			<0.001
MS1	98	16	
MS2	16	57	
MS3	23	200	
**Boyault's Classification**			<0.001
G1/2	52	82	
G3	78	42	
G5/6	6	98	
**Chiang's Classification**			<0.001
Proliferation	109	14	
CTNNB1	7	66	
Interferon	8	67	
Polysomy 7	6	57	
Unannotated	7	29	

Supplementary Table 3. The gene expression of the five-tyrosine metabolism-related genes in HCC cell lines from the Cancer Cell Line Encyclopedia (CCLE) database.

HCC Cell Lines	Gene Expression (log2(TPM+1))				
	ADH4	*GSTZ1*	*ADH1A*	*METTL6*	*LCMT1*
JHH6	0.01	3.63	0.00	4.32	4.32
SNU398	0.01	4.85	0.00	4.76	4.76
SNU886	0.00	4.99	0.00	4.33	4.33
SKHEP1	0.00	3.21	0.00	4.38	4.38
HLF	0.00	3.22	0.00	5.24	5.24
SNU449	0.00	4.53	0.08	4.54	4.54
SNU475	0.06	4.11	0.10	4.28	4.28
LI7	0.00	3.37	0.03	4.45	4.45
HUH1	1.61	2.40	0.20	4.08	4.08
JHH4	0.00	4.31	0.12	4.78	4.78
SNU387	0.00	3.11	0.00	4.96	4.96
HUH7	0.30	3.60	0.76	4.47	4.47
SNU182	0.00	3.75	0.00	5.31	5.31
SNU423	0.00	4.26	0.00	5.27	5.27
SNU761	0.04	5.03	0.01	5.10	5.10
JHH2	0.00	5.33	0.00	5.02	5.02
JHH1	0.23	4.82	0.00	4.67	4.67
HEP3B217	1.59	4.51	0.04	4.36	4.36
HUH6	0.49	4.24	0.00	4.49	4.49
SNU878	3.18	3.89	0.34	4.11	4.11
JHH5	3.41	3.97	0.29	3.98	3.98
HEPG2	5.28	3.89	0.10	5.01	5.01
JHH7	0.64	3.79	0.06	4.66	4.66
PLCPRF5	0.26	4.32	0.75	4.78	4.78

Supplementary Table 4. The drug sensitivity of HCC cell lines from the Genomics of Drug Sensitivity in Cancer (GDSC) platform.

HCC Cell Lines	IC50			
	5-Fluoroucil	Oxaplatin	Irinotecan	Sorafenib
C3A	279.989989	53.532567	69.620838	12.808221
Hep3B2-1-7	38.725098	27.925229	17.471516	3.646564
HLE	153.958218	265.066022	7.390911	12.834059
huH-1	522.572679	183.766803	52.191645	16.563845
HuH-7	1869.482765	529.995809	370.867438	4.168413
JHH-1	1138.280954	148.696271	156.606423	12.856761
JHH-2	571.89069	511.977994	46.671489	21.709979
JHH-4	1306.995746	410.269122	18.345116	28.647883
JHH-6	2592.599909	178.125195	112.56424	44.172739
JHH-7	707.738186	549.777785	279.6123	16.453898
SK-HEP-1	202.699985	180.990643	23.964866	9.810282
SNU-387	433.282383	347.789335	279.21391	334.82217
SNU-398	68.901388	64.499923	12.600223	7.746613
SNU-423	80.841383	491.07997	31.072077	60.955214
SNU-449	417.739204	137.377404	1470.689273	71.007562

IC50, 50% inhibiting concentration.
